# Jet-Black Eschar

**DOI:** 10.3201/eid0808.020171

**Published:** 2002-08

**Authors:** Janet R. Gilsdorf

**Affiliations:** Ann Arbor, Michigan

At first glance, the baby appeared healthy. She was pudgy, pink-cheeked, and impeccably clean and rested quietly in the mother’s arms—until we disturbed her; then she cried with the weary wail of an infant hurting for days. 

“This one came first,” said the mother, pointing to a quarter-sized red, swollen nodule to the right of the baby’s spine. Embedded in its center was a jet-black eschar the size of a pea. I prodded the edge of the nodule; the baby curled her legs, buried her head in the mother’s shirt, and screamed. “There are more,” continued the mother, a pretty young woman with an anxious expression, pointing at the other nodules—one on the occiput, one on the labia majora, one on the left upper chest. Two of these also had black eschars. 

Our first questions were routine and probably anticipated by the mother: When did the fever start? How high had the fever been? When did the lumps appear? What medicines had the baby received? The mother carefully answered each question, underscoring her responses with layers of detail, struggling to remain composed. As she spoke, each word, each gesture, each facial expression carried an air of earnestness, reinforcing her belief that the answers would help make the baby well again. While the mother gently rocked back and forth in the examining-room chair, the infant clung to her chest as fiercely as a wide-eyed baby lemur clings to his mother’s furry belly.

The questions that followed were more circumspect and, to the mother, probably puzzling and far removed from the immediate problem of her feverish baby with the lumps. Still, she answered each one completely, dragging information out of her memory as if it were buried treasure. How much did the baby weigh at birth? What was her medical history? Had anyone at home been ill? Where had she traveled? 

We were ready to zero in on possible diagnoses. The next set of questions must have seemed truly absurd to the mother. Another person, one not so engrossed in the well-being of the baby, might have thought our inquiries weird or intrusive, or even trivial or irrelevant: Do you have any spiders at your house? Have you seen bugs crawling on the baby? Has she been in a hot tub? Are you and the baby’s father blood relatives (first or second cousins)? How old was the baby when the umbilical cord fell off?

 Then, I reached for the bioterrorism protocol from the state health department, which prompted a new set of questions. Has the baby been in contact with imported rugs or animal hides? Has she been in contact with anyone who works on a ranch or with livestock? And finally the question we didn’t ask until last winter: Has the baby been in contact with anyone (grandparents, family friends, babysitters) who works at a mail-sorting facility? The mother’s face tensed with bewilderment and disbelief. But, again, she answered carefully and thoughtfully, persevering as we filed through our differential diagnoses. 

A biopsy of the neck nodule was sent for culture and histologic testing. The baby was admitted to the pediatric ward and was started on piperacillin/tazobactam and gentamicin treatment. The next day, the pathology report came back. The lesion was consistent with ecthyma gangrenosum. Large numbers of bacilli were observed, none with the morphologic features of anthrax. The culture report confirmed *Pseudomonas aeruginosa.*

